# Quantitative evaluation of 4D Cone beam CT scans with reduced scan time in lung cancer patients

**DOI:** 10.1016/j.radonc.2019.03.027

**Published:** 2019-07

**Authors:** Abigail Bryce-Atkinson, Thomas Marchant, John Rodgers, Geoff Budgell, Alan McWilliam, Corinne Faivre-Finn, Gillian Whitfield, Marcel van Herk

**Affiliations:** a Division of Cancer Sciences, School of Medical Sciences, Faculty of Biology, Medicine and Health, The University of Manchester, UK; b The University of Manchester, Manchester Academic Health Science Centre, The Christie NHS Foundation Trust, UK; c Christie Medical Physics and Engineering, The Christie NHS Foundation Trust, Manchester, UK; d The Children’s Brain Tumour Research Network, The University of Manchester, Royal Manchester Children’s Hospital, UK; e Radiotherapy Department, The Christie NHS Foundation Trust, Manchester, UK

**Keywords:** Image guided

## Abstract

**Purpose:**

Image guided radiotherapy (IGRT) based on respiration correlated cone-beam CT (4D-CBCT) provides accurate tumour localisation in lung cancer patients by taking into account respiratory motion when deriving setup correction. However, 4D-CBCT scan times are typically longer than for acquisition of 3D-CBCT scans, e.g. 4 min. This work aims to quantitatively evaluate the effect of reduced scan times on 4D-CBCT image quality and registration accuracy in lung cancer patients.

**Methods and materials:**

Scan times down to 1 min were simulated by retaining only projection images corresponding to every second, third or fourth respiratory cycle in forty-four 4D-CBCTs from 15 lung cancer patients. In addition twenty 2-minute scans were acquired for 12 lung cancer patients. Image quality was quantified by assessing registration accuracy in the shorter scan times, comparing to the 4-minute scan registration result where available as reference.

**Results:**

Use of 2-minute scans had little impact on registration accuracy or ability to detect tumour motion: automatic registration accuracy was within 2 mm in 6/8 scans analysed with 2-minute acquisitions, and 96.6% of registration discrepancies were within 2 mm for the simulated scans. When the scan time simulated was below 2 min, automatic registration results still agreed within 2 mm for 84.7% of scans, however visual image quality was considerably degraded.

**Conclusion:**

A 4D-CBCT acquisition time of 2 min produces scans of sufficient image quality for IGRT in most lung cancer patients, as demonstrated quantitatively by assessing the impact on automatic registration accuracy in simulated and real acquisitions.

## Highlights

• Fast (2 min) 4D CBCT can be simulated accurately from long (4 min) scans.
• Registration was accurate for 96.6% of simulated 2 min scans.
• Acquired 2 min scan registration was accurate in 6/8 patients.
• 2 min 4D CBCT produces sufficient image quality for IGRT in lung cancer patients.

Accurate patient setup in radiotherapy is achieved through the use of cone beam CT (CBCT) image guidance to derive setup corrections to be applied prior to treatment delivery [1], [2]. Organ motion due to respiration is present in the lung; therefore, CBCT projections correspond to different breathing phases. This leads to artefacts (blurring), when scans are reconstructed in 3D [3], [4], [5]. A solution to this issue is to use 4D-CBCT, where projection images are sorted into several respiratory phase bins (typically 10). Such 4D reconstructions reduce motion blurring and allow visualisation of the tumour trajectory over the breathing cycle [4]. In 4D-CBCT, a small number of projections are used to reconstruct each phase. This leads to poorer image quality than 3D-CBCT with streak artefacts due to large angular gaps between projection images [6], [7], [8], [9]. Therefore, longer acquisition times are used than in 3D-CBCT. For example, when using the recommended Elekta XVI lung IGRT protocols (Elekta Oncology Systems, Stockholm, Sweden), the typical acquisition time for a 4D-CBCT is 4 min compared to 1–2 min for a 3D-CBCT [5], [10].

Although 4D-CBCT provides more accurate tumour localisation, there are disadvantages to the longer acquisition time. Patient throughput is reduced and the likelihood that the patient will move before delivery of radiotherapy (intrafraction motion) is increased, particularly for stereotactic treatments (SABR) where the total time on the couch can exceed 30 min [11], [12], [13], [14].

Previous optimisation studies used phantom measurements to quantify the reduction in image quality due to dose and scan time reduction, assessing factors such as contrast to noise ratio (CNR) and spatial resolution [15], [16], [17], [18], [19]. Santoso et al [18] recommend that image quality, dose and reconstruction time are optimal for a scan time of between 2–3 min. A similar conclusion is drawn by Kember et al [20], who performed a visual grading analysis to show that a scan time of 2 min and 13 s yielded a scan of sufficient image quality for use in IGRT. However, none of these evaluations quantitatively assessed the effect on setup, which is specific to the IGRT task.

Image quality can be assessed quantitatively for the task of image guidance, namely tumour localisation, by comparing the results of automatic image registration on images reconstructed with different settings [10], [14], [21], [22]. Rit et al [10] compared the results of motion correction for 3D vs 4D-CBCT scan methods using previously acquired patient data, quantifying the accuracy of each method by comparing image registration results. Ahmad et al [14] used the results of automatic image registration to assess the tumour motion trajectories in a 4D phantom and a single patient for simulated shorter scan time by discarding projection images prior to reconstruction.

The aim of this study was to quantify the accuracy of tumour localisation in 4D-CBCT acquired with shorter scan times for multiple patient cases. For this purpose, we (1) retrospectively simulated shorter scan times from regular 4D-CBCT scans of lung cancer patients and (2) analysed scans acquired with shorter scan times. For both datasets, we assessed the impact on registration accuracy and tumour motion detection.

## Methods and materials

Approval for the use of data was granted by the UK Computer Aided Theragnostics (ukCAT) Research Database Management Committee (based at The Christie NHS Foundation Trust). Research Ethics Committee (REC) approval for the ukCAT Research Database was issued by the North West REC – Haydock on 28/02/2017 (REC reference: 17/NW/0060).

A total of 67 4D-CBCT scans from 20 lung cancer patients were selected retrospectively from data acquired as part of a previous clinical trial (conventional radical radiotherapy patients 01–08) [23] and from scans routinely acquired in clinic (SABR patients 09–20) using an Elekta XVI system. The Elekta recommended 4D-CBCT preset acquires approximately 1320 projection images at 120kVp, 20 mA, and 16 ms per image over a 200 degree gantry rotation with a gantry speed of 50 degrees per minute. These scans take 4 min to acquire, with a nominal scan dose (CTDI_w_) of 11.8 mGy. Patients with a tumour motion vector length of 5 mm or greater (measured from the 4D tumour registration of the first 4D-CBCT acquired) and patients undergoing SABR treatments were selected (Table 1). Average breathing cycle length was calculated by counting cycles from the plotted respiratory signal or from the Amsterdam shroud image generated during reconstruction [24].

**Table 1:** Patient characteristics for Patients 01–20 detailing tumour motion amplitude, ITV, tumour location, tumour stage, breathing cycle length and treatment type. *Patient 13 was excluded from analysis due to failure of the scan to reconstruct in 4D for both 4-minute and 2-minute scans.

Patient	Tumour motion amplitude (mm)	ITV (cc)	Tumour Location	T Stage	Average Breathing cycle length (s)	Treatment type
Pt01	5.50	12.02	Left Lower Lobe	1	3.70	Conventional
Pt02	7.60	48.08	Right Upper Lobe	3	4.64	Conventional
Pt03	6.60	27.67	Left Upper Lobe	2	3.69	Conventional
Pt04	7.40	217.98	Left Lower Lobe	3	3.87	Conventional
Pt05	5.30	21.24	Right Upper Lobe	2	3.86	Conventional
Pt06	6.20	44.42	Left Upper Lobe	3	3.63	Conventional
Pt07	7.00	33.76	Left Lower Lobe	2	3.49	Conventional
Pt08	10.20	18.53	Right Lower Lobe	2	3.73	Conventional
Pt09	15.60	18.10	Right Middle Lobe	2	4.70	SABR
Pt10	18.70	11.60	Right Middle Lobe	1	3.79	SABR
Pt11	11.30	6.40	Left Lower Lobe	1	3.34	SABR
Pt12	16.80	5.60	Left Lower Lobe	1	3.97	SABR
Pt13*	-	4.90	Left Upper Lobe	1	-	SABR
Pt14	9.28	12.1	Right Lower Lobe	1	3.88	SABR
Pt15	10.40	8.70	Right Middle Lobe	1	4.68	SABR
Pt16	11.79	10.00	Right Middle Lobe	1	3.16	SABR
Pt17	5.10	5.00	Right Lower Lobe	1	2.76	SABR
Pt18	11.87	5.00	Right Lower Lobe	1	2.83	SABR
Pt19	10.06	3.40	Right Lower Lobe	1	3.63	SABR
Pt20	12.79	2.50	Right Lower Lobe	1	4.34	SABR
Averages	9.97	–	–	–	3.77	SABR

Reduced scan time was simulated from forty-four 4-minute scans (15 patients) by discarding projection images corresponding to selected respiratory cycles prior to reconstruction using Elekta XVI software [14]. Complete projection data were not available for all scans, so this method could not be applied to all patients. Three shorter scan times were simulated: 2 min, 1 min 20 s and 1 min. Respiratory cycles were removed in an alternating fashion by keeping either the 1st/2nd respiratory cycle out of 2, keeping the 1st /2nd /3rd respiratory cycle out of 3, or keeping the 1st/2nd/3rd/4th respiratory cycle out of 4 respectively. Alternating cycles were sampled from the full 200 degree gantry range. This emulated a faster gantry rotation since complete respiratory cycles were captured, rather than discarding the final 100 degrees of gantry angles or discarding alternate projection images, which would undersample projection angles within the individual breathing cycles.

Twenty 2-minute scans were acquired between 12 patients (Patients 09–20). For 9 of these patients, a 2-minute and 4-minute scan were acquired in a single fraction, either with a 2-minute post-correction scan to verify the applied couch shift derived from the standard 4-minute CBCT; or when a 2-minute scan was used initially but judged on treatment as insufficient to localise the tumour so a 4-minute scan was subsequently acquired. The remaining 3 patients had 2-minute scans acquired only. The 2-minute preset used was identical to the standard 4-minute preset, except with a doubled gantry speed of 100 degrees per minute, acquiring approximately 660 projections with nominal scan dose (CTDI_w_) of 5.9 mGy.

Once reconstructed, each scan was automatically registered to a reference CT to derive a setup correction using a dual registration technique. Although the average reconstruction of the planning 4D-CT is used as the reference scan clinically in our centre, a motion compensated scan was generated and used as the reference CT, removing blurring artefacts from respiratory motion and resulting in sharper definition of the tumour [25]. This scan was unavailable for Pt01, so the average planning 4D-CT was used.

Dual registration was performed using the Elekta XVI software in two stages: (1) a rectangular region of interest (ROI) placed over the bony anatomy of the spine used to perform a greyscale value match (translation and rotations), and (2) a 4D mask greyscale value match on a ROI centred on the tumour (translation only). The couch shifts were derived from the tumour mean position from each registered frame. The mask for 4D registration comprised of the ITV + 5 mm margin, edited manually using a paintbrush tool to remove any nearby bony anatomy from the ROI. Greyscale value match was used for registration of the clipbox region to draw comparison between all simulated short scan times, since reconstruction of the 1 min 20 s and 1 min scan times provided insufficient scan quality for the bone matching algorithm. Once reconstructed, the image quality of each scan was inspected visually and quantitatively by comparison of the automatic registration results.

Since the simulated scans were created from the original scan, there were no differences in positioning or breathing rate from the original 4-minute scan. Therefore a direct comparison between couch shifts could be made, using the shifts derived from the 4-minute scan as gold standard. Automatic registration results were compared by calculating the vector differences in the derived couch shifts (mean tumour position) and the bone position between each simulated scan and the 4-minute scan. The 4D tumour registration was also compared at each phase to assess the effect of short scan time on tumour motion detection. All data are presented in the superior–inferior (SI), left–right (LR), and anterior–posterior (AP) directions.

The actual 2-minute scans were a different acquisition to the 4-minute scans, meaning that a direct comparison of couch shifts could not be made as with the simulated scans. Instead, an offset between the bone and average tumour match was calculated for each scan. This dual registration offset was expected to be similar between scans from the same patient if the registration was performed with equivalent accuracy. Standard deviations in the offset measurements were calculated for patients with multiple 2-minute acquisitions.

For scans where 2-minute and 4-minute scans were acquired in a single fraction, the tumour match was additionally assessed to look at the amplitude and trajectory of the tumour motion. The differences in tumour match in LR, SI and AP directions were compared between the 2 and 4-minute scans for each phase.

## Results

**Fig. 1:**
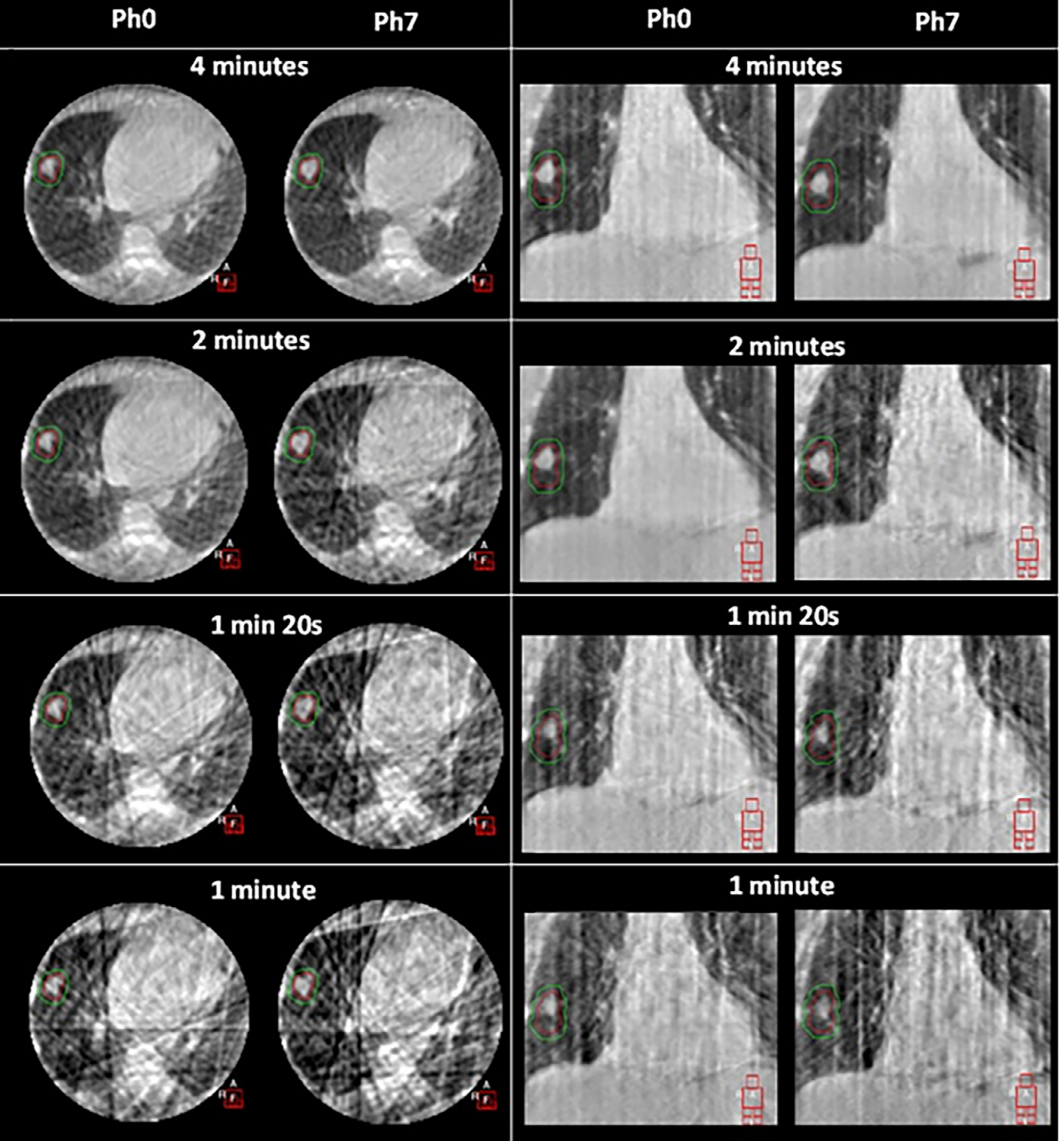
Comparison of visual image quality for Patient 02 showing axial (left) and coronal (right) slices at peak exhale (phase 0) and mid exhale (Phase 7) to illustrate phases with best and worst image quality for each simulated scan time.

The image quality of the simulated shorter scans is shown in Fig. 1. Image quality degraded as the scan time became shorter, with a greater appearance of streak artefacts and image noise. Tumour motion could still be observed even at the shortest length scans; however, the poorer image quality made differentiation of the tumour and lung tissue more challenging.

Dual registration accuracy results for the simulated shorter scans are shown in Table 2. 4D registration remained very accurate for simulated 2-minute scans: 96.6% had 4D tumour registrations within 2 mm. Bony anatomy registrations remained accurate for all simulated scan times, with all 2-minute scan bone registration discrepancies within 2 mm. The frequency and magnitude of discrepancies increased as scan time decreased. Image registration was deemed failed if the couch shift discrepancy was greater than 2 mm.

**Table 2:** Accuracy of automatic registration for each simulated short scan time compared to the 4-minute scan. Reported discrepancies are vector lengths.

	Derived couch shift (mean tumour position)			3D Bone match (Clipbox registration)			4D Tumour match (across every phase)		
Simulated scan time	2 min	1 min 20 s	1 min	2 min	1 min 20 s	1 min	2 min	1 min 20 s	1 min
Scans with discrepancy < 2 mm	98.9%	95.5%	93.8%	100%	99.2%	98.9%	96.6%	88.6%	81.8%
Mean discrepancy (mm)	0.29	0.47	0.63	0.30	0.41	0.55	0.24	0.34	0.45
No. failures	1	6	11	-	-	-	-	-	-

**Fig. 2:**
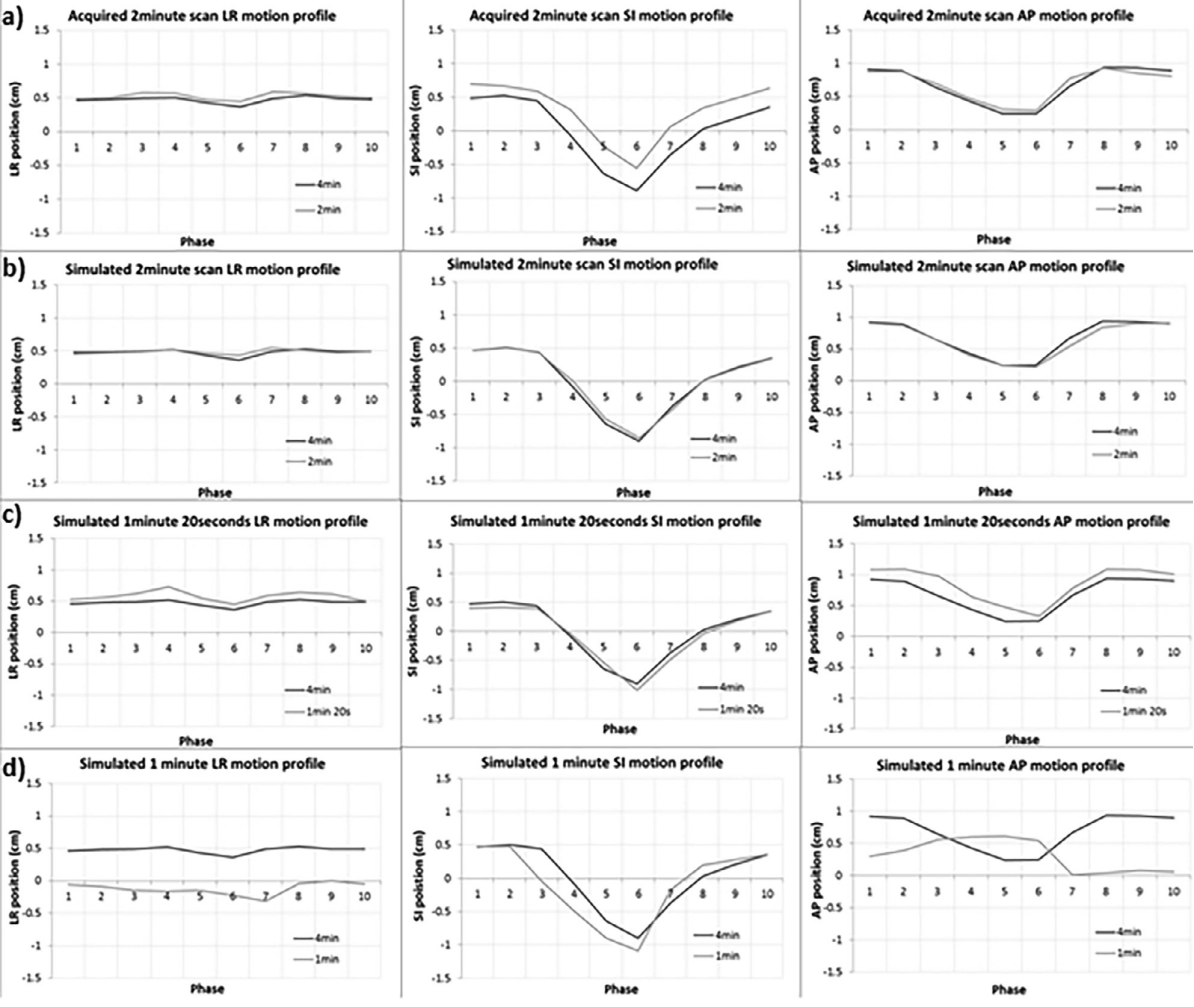
Tumour motion profiles for Patient 09 comparing the acquired 2-minute scan (a) and simulated 2-minute (b), 1 min 20 s (c) and 1 min (d) scans against the 4-minute scan. The acquired 2-minute scan data are offset by the applied couch shifts between scans such that the profiles can be compared.

Fig. 2b-d shows the tumour motion profile for Pt09, comparing 4-minute scans to simulated shorter scan times. The shapes of the profiles and registration at each phase are almost identical for the 4-minute and simulated 2-minute scan, but differences increase as the scan time becomes shorter.

**Fig. 3:**
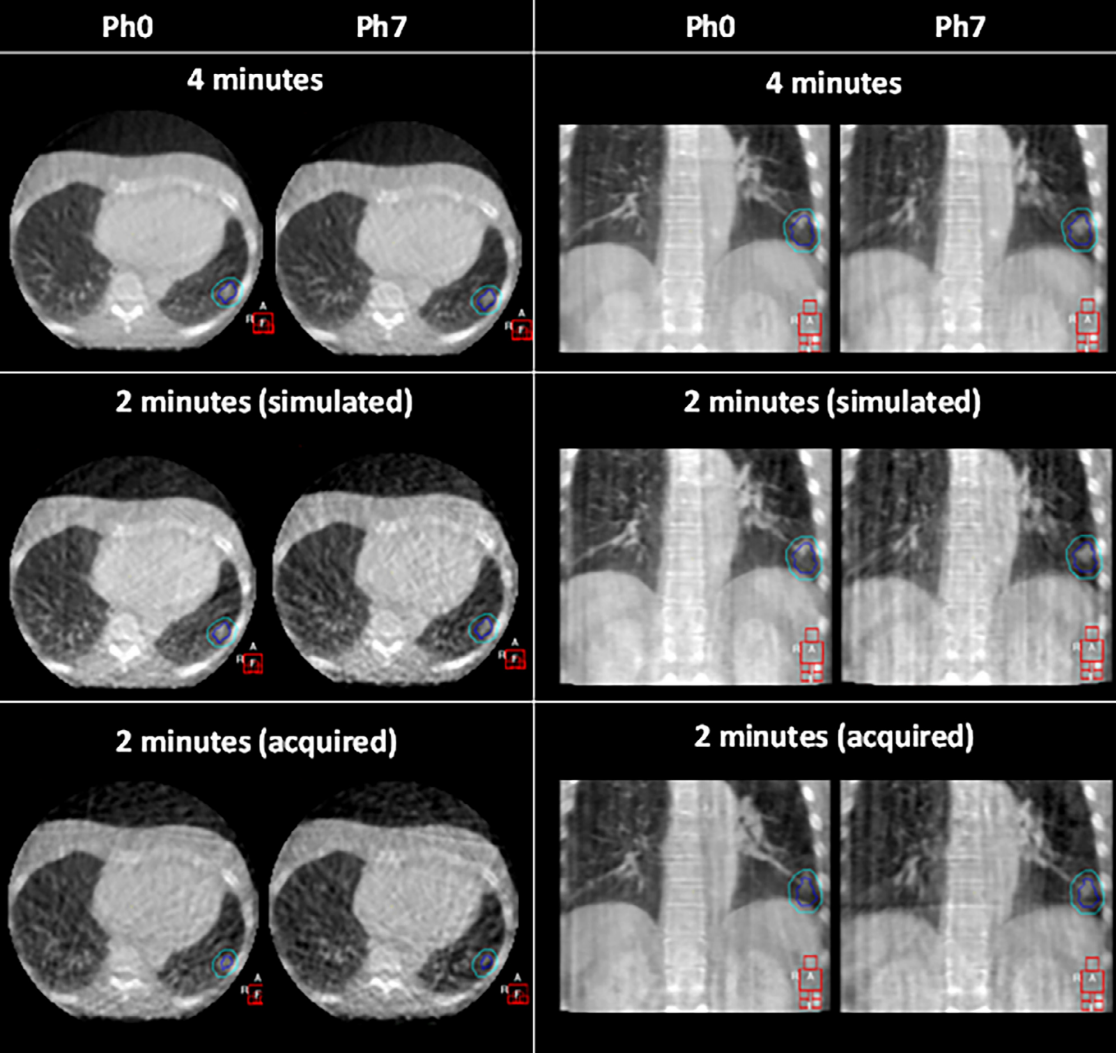
Visual comparison of the acquired 2-minute and simulated 2-minute scans with the original 4-minute scan for phases 0 and 7 in axial (left) and coronal (right) view for Patient 11.

Fig. 3 compares the visual image quality of a 4-minute, simulated 2-minute and acquired 2-minute scan for Patient 11. The simulated and acquired 2-minute scan showed similar image quality, with noise and streak artefacts more apparent than in the 4-minute scan. In spite of this, the tumour could still be clearly identified in most breathing phases. Tumour motion is generally clearer in the 4-minute scans, however, the motion could still be observed in the 2-minute scans. This comparison served as a simple validation that the simulation method accurately depicted a scan acquired with a shorter scan time.

Fig. 2a shows the tumour motion profile of Patient 09. The 2-minute acquisition profile was offset by the values of the couch shift between scans such that the position at each phase can be compared relative to the 4-minute scan position. The tumour motion profiles were almost identical, with a baseline shift shown in the SI direction.

Table 3 shows the comparison between dual registration offsets for all acquired 2-minute scans. Patient 13 was excluded from this analysis since both 4-minute and 2-minute 4D-CBCT scans had artefacts due to the patient’s chin being in the field of view and almost no tumour motion, which prevented 4D reconstruction; therefore 3D scan were used during treatment. Dual registration offsets were consistent within 2 mm for 6/8 patients with same-fraction scans. Vector differences of greater than 2 mm could be attributed to poor scan quality for both the 2-minute and 4-minute scans. Patients who received 2-minute scans over multiple fractions showed consistency between dual registration offsets, with all standard deviations within 2 mm in all directions.

**Table 3:** Registration accuracy for the acquired 2-minute scans for Patients 09–20, detailing comparisons of dual registration offsets and % phases with discrepancies greater than 2 mm compared to the 4-minute scans.

Patient	Scan time (minutes)	Dual registration offset (mm)			Vector difference (mm)	%phases with discrepancies > 2 mm		
		LR	SI	AP		LR	SI	AP
09	4	2.00	−1.60	4.10	1.97	0	80	0
	2	2.00	0.20	4.90				
10	4	−0.20	1.70	0.90	1.32	0	0	0
	2	0.00	0.50	0.40				
11	4	−2.20	−2.30	−0.30	1.40	30	0	0
	2	−1.70	−3.40	−1.00				
12	4	0.50	−4.30	−0.40	1.95	10	10	0
	2	−0.90	−3.50	−0.70				
14	4	−0.80	−0.10	−0.40	1.69	0	80	0
	2	0.60	−0.40	−1.30				
15	4	−2.70	4.00	6.70	4.01	100	100	100
	2	−1.30	7.40	8.30				
18	4	1.00	−0.50	−1.90	1.21	0	0	0
	2	0.50	−1.70	−2.60				
20	4	0.20	−2.00	0.90	14.08	100	100	100
	2	3.30	10.10	−5.60				

Since clinical implementation, a total of 42 patients (213 CBCTS) were scanned using the 2-minute scan protocol. Only 8 patients were required to revert back to the 4-minute scan protocol. Patients who reverted to 4-minute scans continue to receive 4-minute scans for the remainder of treatment, whereby 17.8% (38 CBCTs) of total CBCT sessions were 4-minute scans. The average breathing cycle for these patients was 4.37 s with standard deviation 0.76 s. The majority of scans that reverted to 4-minute scans were during the initial 4 months of implementation, suggesting that radiographer confidence in accepting the 2-minute scans increased as they adjusted to the differences in visual image quality. Common reasons for reverting to a 4-minute scan time included: difficulty visualising the target due to congestion in the lungs and poor visualisation of tumours that were close to the diaphragm or chest wall.

## Discussion

4D-CBCT scan times shorter than the standard 4-minute 4D-CBCT scans have been simulated in 15 patients with a range of tumour amplitudes. A 2-minute 4D-CBCT scan preset has been evaluated in 12 patients and the results validate the simulation method. The work shows quantitatively that halving the scan time to 2 min produces 4D-CBCT scans of sufficient quality for use in IGRT of lung cancer patients (eg SABR IGRT), extending on previous work involving phantoms or single patient studies [14], [18], [19].

The registration discrepancy between 2-minute and 4-minute scans was greater than 2 mm in 2/8 patients. This method relies on the 4-minute scan as a gold standard for comparison; however some unreliability is therefore introduced for when 4-minute scans are of poorer quality. The registration discrepancy for patient 20 exceeded 1 cm and could be attributed to poor definition of the target during the inhalation phase in both the 2-minute and 4-minute scan. In this case the patient received a repeat 4-minute scan where image quality was seen to improve. It is therefore important to note that patient dependent factors such as discomfort, position inaccuracy and breathing irregularities degrade image quality and are of considerable importance due to longer scan times [5]. Comparison of the simulation method to the acquired 2-minute scans showed an accurate representation of the image artefacts introduced due to sparse projection images and is therefore a useful tool for this analysis.

This quantitative data are in good agreement with the results of the visual grading analysis study by Kember et al [20], also recommending the use of a 2-minute scan time. The preset presented in this study differs from Kember et al since exposure settings per frame were unchanged, thus dose is also halved. Increasing the exposure could improve the visual image quality; however, the major artefact in the shorter scan times is streaking due to limited projections rather than exposure related image noise [18]. The 2-minute acquisition scan time is comparable to 3D imaging, which has been shown to localise the mean tumour position equivalently well as 4D-CBCT [26]. With a reduction in 4D-CBCT scan time to 2 min, the concerns over scan time are alleviated, with the benefit of being able to reconstruct an average CBCT from the data to gain a 3D scan at no extra dose cost.

The 2-minute scans in this study were shown to be of sufficient image quality for the range of breathing rates studied, however limited by the lack of slower breathing patients. The average patient breathing rate in patients reverted to a 4-minute scan was 4.37 s, comparable with the breathing rates measured in the cohort of patients studied. In these patients, recorded clinical observation suggested that poorer image quality was due to congestion in the lungs and tumour location. Previous studies have included adapting gantry speed in real-time to match the patient’s respiratory signal in order to improve image quality. These methods require modification to machine hardware to implement and can result in long scan times predicted for slow breathing patients [16], [17], [27]. This requires additional operator responsibilities to analyse cost–benefit of image quality and scan time on patient specific basis [27]. In our method the 4-minute scan remains an option, and reverting to 4 min scan time would only increase patient dose by a maximum of 1 additional scan in cases in cases where a 4-minute scan was required in the first fraction.

Despite poorer image quality, registration remained accurate even in the majority of the 1 min scans. However, even with accurate registration, the visual image quality must also be good enough for operators to have confidence in the results. Therefore, the 2-minute scans appear to strike the best balance between improving the speed of imaging and maintaining reliable automatic registration and suitable visual image quality. Keeping a short scan time and hence lowering overall treatment time is desirable for frail patient populations such as lung cancer patients treated with SABR. Reducing the patient’s time spent on the treatment couch reduces patient discomfort levels and the likelihood of intrafraction motion.

## Conclusions

Shortening 4D-CBCT acquisition time to 2 min produces scans of sufficient image quality for IGRT. These scans maintain registration accuracy and tumour motion detection in lung cancer patients. This is of clinical benefit as a shorter scan time will reduce the risk of intra-fraction motion and improve patient throughput in the clinic, whilst halving dose to the patient. Two minute 4D-CBCT scans have been validated here in 12 patients and are now being implemented in our hospital with the 4-minute scan remaining as an option where improved image quality is required.

## Sources of support

This work was supported by CRUK via the funding to Cancer Research UK Manchester Centre: [C147/A18083] and [C147/A25254]. Prof. van Herk was supported by NIHR Manchester Biomedical Research Centre. Dr Budgell reports grants and non-financial support from Elekta, outside the submitted work. Prof. van Herk reports grants from Elekta Oncology Systems, during the conduct of the study and has a patent 4D Cone beam CT with royalties paid to Netherlands Cancer Institute.

## Conflict of interest

None.
